# Enrichment and Purification of Syringin, Eleutheroside E and Isofraxidin from *Acanthopanax senticosus* by Macroporous Resin

**DOI:** 10.3390/ijms13078970

**Published:** 2012-07-18

**Authors:** Fengjian Yang, Lei Yang, Wenjie Wang, Yang Liu, Chunjian Zhao, Yuangang Zu

**Affiliations:** State Engineering Laboratory for Bio-Resource Eco-Utilization, Northeast Forestry University, Harbin 150040, China; E-Mails: yangfj@nefu.edu.cn (F.Y.); wjwang225@hotmail.com (W.W.); yllynefu@163.com (Y.L.); zcjsj@163.com (C.Z.)

**Keywords:** Radix *Acanthopanax senticosus*, macroporous resin, syringin, eleutheroside E, isofraxidin

## Abstract

In order to screen a suitable resin for the preparative simultaneous separation and purification of syringin, eleutheroside E and isofraxidin from *Acanthopanax senticosus*, the adsorption and desorption properties of 17 widely used commercial macroporous resins were evaluated. According to our results, HPD100C, which adsorbs by the molecular tiers model, was the best macroporous resin, offering higher adsorption and desorption capacities and higher adsorption speed for syringin, eleutheroside E and isofraxidin than other resins. Dynamic adsorption and desorption tests were carried out to optimize the process parameters. The optimal conditions were as follows: for adsorption, processing volume: 24 BV, flow rate: 2 BV/h; for desorption, ethanol–water solution: 60:40 (v/v), eluent volume: 4 BV, flow rate: 3 BV/h. Under the above conditions, the contents of syringin, eleutheroside E and isofraxidin increased 174-fold, 20-fold and 5-fold and their recoveries were 80.93%, 93.97% and 93.79%, respectively.

## 1. Introduction

Radix *Acanthopanax senticosus* (RAS) consists of the dried roots and rhizomes of *Acanthopanax senticosus* (Araliaceae) [1]. RAS has been used extensively in China, Russia, Korea and Japan as an adaptogen [2,3]. Nowadays, there are some RAS products, including drugs and health food, on the market in many countries [3,4]. In China, it is common for people using RAS to make medicinal liquor or to stew pigs, milk cows and pigeons. *In vitro* and *in vivo* studies have demonstrated that RAS possesses many pharmacological effects, such as antistress, antifatigue, immunoenhancing, antidepressive effects, *etc.* [4,5].

According to previous research results, syringin, eleutheroside E and isofraxidin are the major components attributed to the pharmacological effects of RAS. Syringin (also termed eleutheroside B) was reported to exhibit an antifatigue effect [6] and immunomodulatory activity [7]. Further investigations indicate that syringin is also useful for releasing acetylcholine, increasing insulin secretion [8], decreasing sympathetic tone in conscious animals [9] and improving neurite outgrowth [10]. It has been shown that eleutheroside E possesses an antistress [11] and anti-fatigue effect [4,11]. Meanwhile, several studies have suggested that isofraxidin has antibacterial [12] and antiinflammatory effects [13].

Although syringin, eleutheroside E and isofraxidin in RAS each contribute to different pharmacological activities, they are the most important qualitative standards for testing RAS products. Syringin and eleutheroside E are used for RAS’s quantitative determination by the World Health Organization [1]. Meanwhile, in the Chinese Pharmacopoeia (2010 edition), isofraxidin is required as the quantitative parameter of RAS [14]. In other words, it is necessary for an adaptogen made of RAS to contain all of the three components. Thus, for economical interests, it is very important to separate and purify syringin, eleutheroside E and isofraxidin from RAS.

Many methods have been used to effectively separate components from RAS, such as liquid–liquid partition [15], column chromatographic procedures involving silica gel [16] or polyamide [17] and centrifugal partition chromatography [18]. However, due to their relative low handling capacity in one cycle, these separation methods are inefficient. They are also troubled with various other disadvantages, such as low recovery, large solvent consuming, high labor intensities and operation cost, and even safety problems. Those deficits make them unsuitable for large-scale industrial production [19,20]. As a kind of adsorbent, macroporous resin can be used to selectively adsorb constituents from aqueous solutions through hydrogen bonding forces and van der Waals forces, *etc.* [21,22]. These forces are based on differences in pore structure and surface functional groups of macroporous resin [20], as well as, amongst others, differences in molecular weight, molecular polarity, and molecular shape of the constituents in aqueous solution. [23]. The adsorption–desorption process by macroporous resins, as an efficient separation method, usually shows a high adsorption, easy desorption, low costs of operation and easy regeneration [24,25]. Furthermore, an ever-growing research field has been studying the use of macroporous resins to separate pharmacological components from herbal materials [20,26–30].

Moreover, in the previous studies [31,32], only a few kinds of macroporous resins were tested for the separation of syringin [32], or syringin and eleutheroside E [31] from RAS extracts, the resin was not optimized and the adsorption capacity was not satisfactory, the parameters for static adsorption and desorption tests were not optimized either. The simultaneous separation and purification of the three targeted components from RAS using a macroporous resin has, to the best of our knowledge, not yet been reported in the literature. This study aims to develop an efficient method for the separation of the three targeted components with the optimal resin. The information in this study could facilitate the selection of suitable macroporous resins for preparative separation and purification of other phenol glycosides and aglucones all at once from other herbal materials in the future.

## 2. Results and Discussion

### 2.1. Adsorption and Desorption Properties of the Resins

As shown in Table 1, the adsorption and desorption properties of different resins for syringin, eleutheroside E and isofraxidin are distinct. The adsorption capacities of HPD100C and HPD300 for the three targeted components are higher than those of other resins. HPD100C and HPD300 have a larger surface area, which may account for their higher adsorption capacities for the three targeted components. Meanwhile, HPD100C and HPD300 are non-polar, while the three targeted components do not belong to compounds of high polarity either. Therefore, due to their high surface area and similar polarity with the three targeted components, HPD100C and HPD300 possess better adsorption capacities.

On the other hand, HPD100C and HPD300 also showed higher desorption capacities and desorption ratios for the three targeted components. This may be due to the fact that the affinity between the components and the resins is mainly based on physical forces, such as the van der Waals force [22,23], which has a low power for holding the three targeted components on HPD100C and HPD300. In sum, the ranking in Table 5 appears to reflect the different physicochemical properties of these resins. Compared with other resins, HPD100C and HPD300 possess better adsorption and desorption capacities and a higher desorption ratio for syringin, eleutheroside E and isofraxidin. Therefore, HPD100C and HPD300 were selected for further study in the following adsorption kinetics experiments.

### 2.2. Static Adsorption Kinetics on HPD100C and HPD300

**Figure 1:**
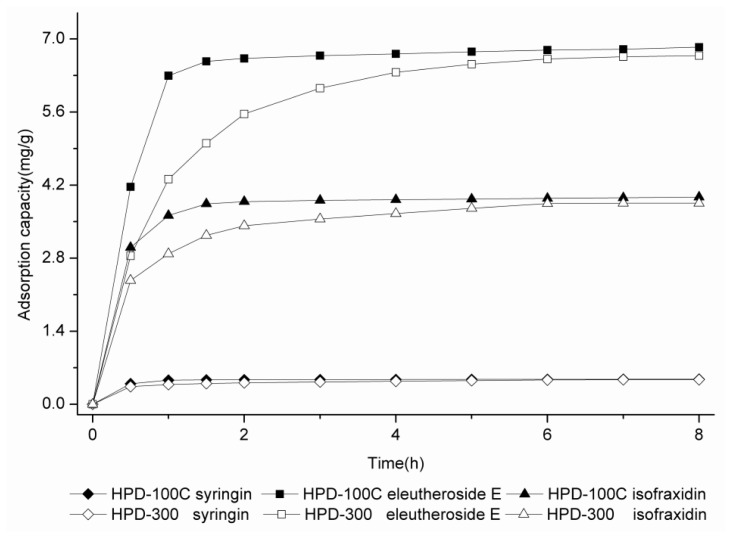
Adsorption kinetics curves for syringin, eleutheroside E and isofraxidin on HPD100C and HPD300.

A more suitable resin must also have a higher adsorption rate. Therefore, the adsorption kinetics curves were obtained for syringin, eleutheroside E and isofraxidin on HPD100C and HPD300. As can be seen in Figure 1, the adsorption capacities of the three targeted components increase with the increase of the adsorption time, reaching an equilibrium at about 2 h on HPD100C and 6 h on HPD300. In Figure 1, it is apparent that HPD100C has a better adsorption rate than HPD300. Thus, HPD100C was selected for further study in the following tests.

### 2.3. Adsorption Isotherms

**Figure 2:**
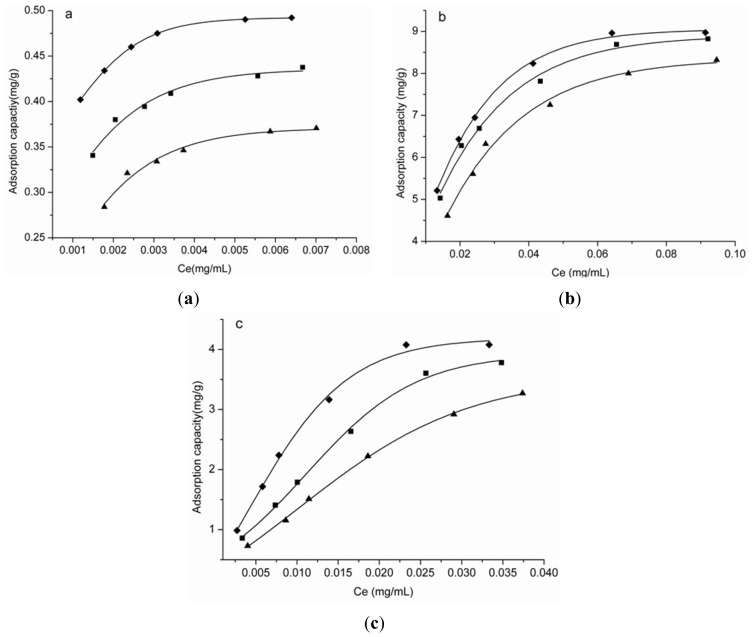
Adsorption isotherm at 25 °C (◆), 30 °C (■) and 35 °C (▲) for syringin, eleutheroside E and isofraxidin on HPD100C. (**a**) Syringin; (**b**) Eleutheroside E; (**c**) Isofraxidin.

The adsorption isotherms of HPD100C for syringin, eleutheroside E and isofraxidin were investigated with different concentrations of sample solutions at 25, 30, 35 °C. The initial concentrations of syringin were 0.0032, 0.0039, 0.0047, 0.0055, 0.0077 and 0.0089 mg/mL; the initial concentrations of eleutheroside E were 0.0390, 0.0518, 0.0590, 0.0824, 0.1090 and 0.1362 mg/mL and the initial concentrations of isofraxidin were 0.0077, 0.0144, 0.0190, 0.0297, 0.0436 and 0.0537 mg/mL, respectively. As shown in Figure 2, the adsorption capacities increase with the increase of the initial solute concentrations, and reach the saturation plateau when the initial concentrations of syringin, eleutheroside E and isofraxidin are 0.0077, 0.1090 and 0.0436 mg/mL, respectively. Thus, these concentrations were used in the following study.

Equilibrium data give information about the affinity between solute and adsorbent. The Langmuir isotherm model and the Freundlich isotherm model are the two best known and most often used isotherm model for the adsorption of solutes from solution.

The Langmuir and Freundlich model-fitting results of the obtained experimental data of the three targeted components are summarized in Table 2.

As can be seen in Table 2, comparing with the fitting results of the Freundlich equation, both syringin and eleutheroside E’s Langmuir fitting equations exhibit higher and more dependable correlation coefficients. Their *R*^2^ values for the Langmuir equation are all above 0.97, but those for the Freundlich equation are much lower than 0.94. Thus, the Langmuir equation is better for describing the adsorption and desorption behavior of syringin and eleutheroside E on HPC100C.

Meanwhile, for the Freundlich equation, the adsorption can easily take place when 1/*n* value is between 0.1 and 0.5, however, this tends not to happen when the 1/*n* value is between 0.5 and 1, and is almost impossible to occur when the 1/*n* value exceeds 1 [27]. In isofraxidin’s Frendlich equation, all 1/*n* values are between 0.5 and 1 (Table 2). Thus, even though the *R*^2^ values of isofraxidin’s Freundlich equation are rather high, it is hard for the adsorption of isofraxidin on HPD100C to happen. On the other hand, the *R*^2^ values of isofraxidin’s Langmuir equation are all above 0.98. Thus, the Langmuir equation can describe the adsorption and desorption behavior of isofraxidin on HPC100C.

The Langmuir isotherm model is based on the assumption that the sorbate form is only a single layer [33], thus the results in Table 2 show that it is the single layer form the adsorption of particles of the three targeted components onto the surface of HPD100C. At the same initial concentration, the adsorption capacities decrease with increasing temperature in the investigated temperature range, which indicates that the adsorption is a thermopositive process. Meanwhile, *Q*_max_ also decreases with increasing temperature for all three targeted components. Therefore, 25 °C was selected in the following experiments.

### 2.4. Dynamic Adsorption and Desorption Tests

#### 2.4.1. Dynamic Breakthrough Curves on HPD-100C Resin

**Figure 3:**
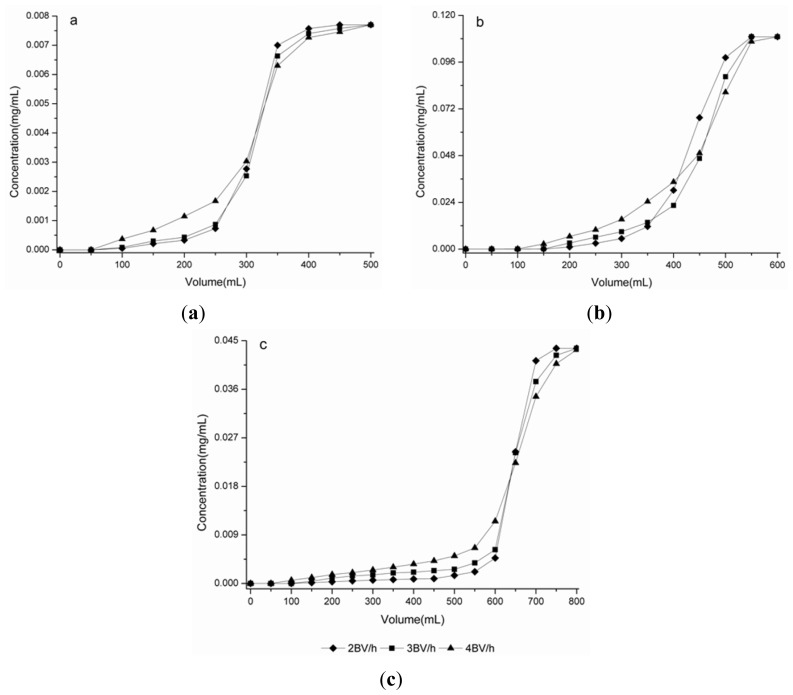
Dynamic breakthrough curves of syringin, eleutheroside E and isofraxidin on columns packed with HPD100C. (**a**) Syringin; (**b**) Eleutheroside E; (**c**) Isofraxidin.

The initial concentrations of syringin, eleutheroside E and isofraxidin in this test were 0.0077, 0.1090 and 0.0436 mg/mL, and the flow rates investigated in this test were 2, 3 and 4 BV/h, respectively. The dynamic breakthrough curves of the three targeted components on HPD-100C resin were obtained based on the effluent volume and the concentration of solute in the sample solution. As can be seen in Figure 3, the three targeted components exhibit better adsorption performance at the flow rate of 2 BV/h, this may be due to a better particle diffusion in the sample solution. Therefore, 2 BV/h was selected in the following tests.

For the breakthrough point, when the concentration in the leak solution is 10% of the initial concentration, adsorption is presumed to have reached saturation. When the adsorption affinity decreases, or even disappears, then the solutes leak from the resin. Thus, the breakthrough point is usually defined as the time when the leak solution concentration is equal to 10% of the initial concentration [19,20,23]. However, due to the different initial concentrations and retention times of the three targeted compounds, they have different breakthrough points. Thus, in order to find out the dynamic breakthrough situation about the three targeted components, it is important to set up the breakthrough curve. In the present case, the breakthrough point of syringin, eleutheroside E and isofraxidin appeared at a processing volume of the sample solution around 10 BV, 14 BV and 24 BV, respectively. The result shows that isofraxidin was eluted much later than syringin and eleutheroside E, which means that if the volume of feed solution was selected according to syringin or eleutheroside E, isofraxidin would not reach adsorption saturation. Therefore, taking all the three targeted components into consideration, a feed solution of 24 BV was selected for dynamic adsorption experiments. At the breakthrough points, the absorption capacities were an amount of syringin of 2.60 mg, eleutheroside E of 49.05 mg and isofraxidin of 29.45 mg.

#### 2.4.2. Effect of Ethanol–Water Solution on Desorption Tests

In order to choose the proper desorption solution, different ethanol–water solutions (30:70, 40:60, 50:50, 60:40, 70:30, 80:20, 90:10, v/v) were used to perform desorption tests for due to its characteristics of being cheaper and non-toxic. As can be seen in Table 3, on one hand, with the increase of the ethanol–water solution, the desorption mass of syringin, eleutheroside E and isofraxidin increases markedly until the ethanol–water solution reaches 60:40 (v/v), and raises slightly past this point; on the other hand, the desorption contents of the three targeted components gradually increase and reach their peak values at the ethanol–water (60:40, v/v) solution, and then decrease. When the ethanol–water solution exceeds 60:40 (v/v), the mass of dried residue is increased, so, the impurities desorbed also increase. At the ethanol–water (60:40, v/v) solution, desorption mass of the three targeted components are 2.10, 46.09 and 27.62 mg, respectively, and their relative contents are the highest compared with those at other ethanol–water solutions. The more desorption contents of the three targeted components, and the smaller the mass of dried residue, the better, thus, ethanol–water (60:40, v/v) solution was selected as the appropriate desorption solution and used in the following dynamic desorption experiments.

#### 2.4.3. Dynamic Desorption Curve on HPD100C

**Figure 4:**
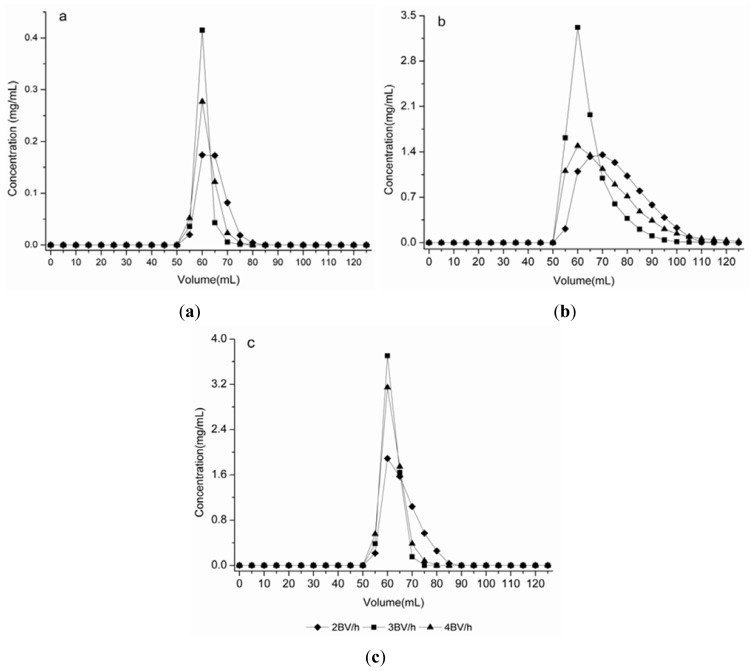
Dynamic desorption curves of syringin, eleutheroside E and isofraxidin on a column packed with HPD100C. (**a**) Syringin; (**b**) Eleutheroside E; (**c**) Isofraxidin.

The ethanol–water (60:40, v/v) solution was used to elute syringin, eleutheroside E and isofraxidin. The flow rates investigated in this test were 2, 3 and 4 BV/h, respectively. The dynamic desorption curves on HPD100C were obtained based on the volume of desorption solution and the concentration of solute. As can be seen in Figure 4, the three targeted components exhibited better desorption performance at the flow rate of 3 BV/h. At this flow rate, syringin was thoroughly desorbed in 3 BV; eleutheroside E in 4 BV, and isofraxidin in 3 BV. The results indicate that the process of 3 BV/h reduces the ethanol solutions used and shortens the desorption process. Therefore, 3 BV/h was selected as the proper desorption flow rate considering its lower volume consumption and high efficiency.

**Figure 5:**
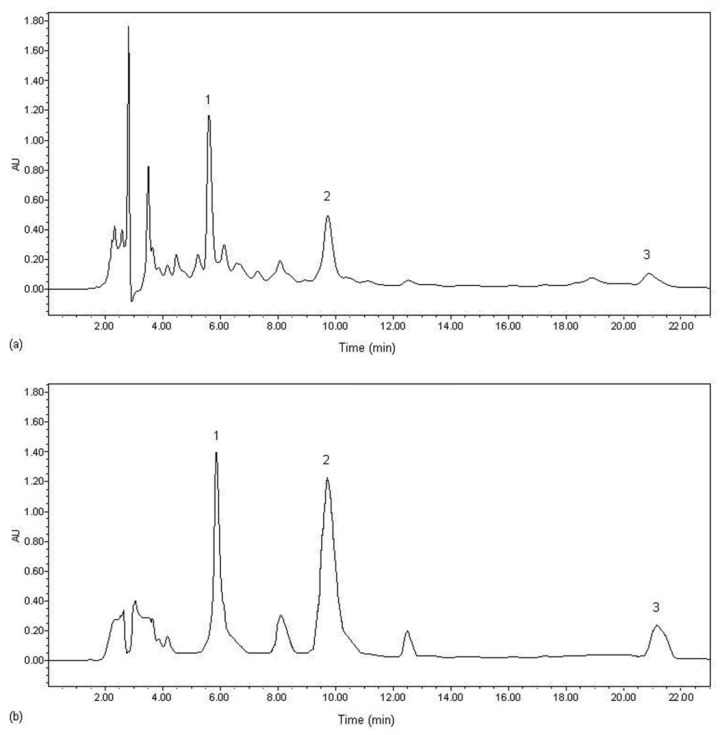
HPLC profiles of sample solution before (**a**) and after (**b**) treated on a column packed with HPD100C. 1. Syringin; 2. Eleutheroside E; 3. Isofraxidin.

The optimum parameters for the preparative separation of the three targeted components on HPD100C were confirmed as follows: for adsorption, the concentrations of syringin, eleutheroside E and isofraxidin in sample solution: 0.0077, 0.1090 and 0.0436 mg/mL, respectively; processing volume: 24 BV; flow rate: 2 BV/h; pH value: 5; temperature: 25 °C for desorption, ethanol–water solution: 60:40 (v/v); eluent volume: 4 BV; flow rate: 3 BV/h. The HPLC profiles of the samples before and after HPD100C chromatography are shown in Figure 5. By comparison, it can be seen that some impurities are removed from the sample solution and the relative peak area of the three targeted components increases pronouncedly after the separation treatment on HPD100C.

The eluate was obtained under optimum conditions, the residual ethanol was concentrated under vacuum by rotary evaporator, and freeze-dried after the ethanol was removed. The contents in the resulting product and the recoveries of syringin, eleutheroside E and isofraxidin were obtained (Table 4).

## 3. Experimental Section

### 3.1. Materials, Chemical and Reagents

Radix *Acanthopanax senticosus* (RAS) was obtained from Sankeshu Medicinal Materials Market (Harbin, China), and then was minced into little pieces and sieved through 20–40 meshes before use. Syringin, eleutheroside E and isofraxidin standard samples (95% purity) were purchased from the National Institute for the Control of Pharmaceutical and Biological Products (Beijing, China). Acetonitrile of chromatographic grade was purchased from J&K Chemical Ltd. (Beijing, China). Deionized water was freshly prepared by a Milli-Q water-purification system (Millipore, Bedford, MA, USA) and used in all experiments. Other reagents were of analytical grade and were purchased from Beijing Chemical Reagents Co. (Beijing, China). All solvents prepared for HPLC were filtered through 0.45 μm nylon membrane and degassed under ultrasonication before use.

### 3.2. Adsorbents

The macroporous resins tested were purchased from Cangzhou Bon Adsorber Technology Co., Ltd. (Cangzhou, China). Their physical properties are listed in Table 5.

In order to remove the monomers and porogenic agents trapped inside the pores of the macroporous resins during the synthesis process, the adsorbent beads were pretreated with the following procedure: First, the resins were soaked in ethanol for 24 h and then washed with deionized water by circumfluence until there was no residue of ethanol. The treated resins were stored in a desiccator with deionized water in order to maintain constant moisture content. Prior to use, the resins were wet with ethanol again and then thoroughly replaced with deionized water. In order to obtain moisture contents of macroporous resins, the resins were accurately weighed in glass dishes, and dried to a constant weight in a digital blast oven (Shanghai Boxun Industry & Commerce Co., Ltd., Shanghai, China) at 105 °C. Their moisture contents are also shown in Table 5.

### 3.3. Preparation of Crude RAS Extracts

The minced RAS powder (2 kg) was extracted with deionized water (12 L) under reflux for 30 min each time [34], this was repeated three times. The extract solution was purified by membrane filtration and then evaporated in rotary vaporization (RE-52AA, Shanghai Huxi Instrument, Shanghai, China) to dryness under vacuum condition. The dry extract was stored at 4 °C. The contents of syringin, eleutheroside E and isofraxidin in the extract were 0.04%, 0.59% and 0.24%, respectively. Deionized water was added to get sample solutions at the concentration range of syringin 0.002–0.020 mg/mL, eleutheroside E 0.025–0.250 mg/mL and isofraxidin 0.006–0.060 mg/mL, respectively.

### 3.4. HPLC Analysis of Syringin, Eleutheroside E and Isofraxidin

A Waters liquid chromatograph (Waters Corporation, Milford, MA, USA), consisting of a Waters 600 Controller equipped with a Waters 717 plus autosampler, and a Waters 2487 UV detector was used to determine syringin, eleutheroside E and isofraxidin. Chromatographic separation was carried out on a Kromasil C18 reversed-phase column (5 μm diameter particles, 4.6 mm × 250 mm I.D., Kromasil). The mobile phase was acetonitrile–water–formic acid (15:84.9:0.1, v/v/v). The detection wavelength was 205 nm, the flow rate was 1 mL/min, the injection volume was 10 μL, and the column temperature was maintained at 25 °C. For standard sample solution, various amounts of syringin, eleutheroside E and isofraxidin were dissolved in methanol to yield the stock solutions at concentrations of 0.9840, 0.1012 and 0.1016 mg/mL, respectively, and the retention times were 5.5, 9.0 and 20.5, respectively. The calibration curves of the three targeted components, that showed good linearity, were *y* = 9 × 10^7^*x* − 158523 (*R*^2^ = 0.9988), *y* = 6 × 10^7^*x* − 416914 (*R*^2^ = 0.9979) and *y* = 2 × 10^7^*x* − 139160 (*R*^2^ = 0.9916), respectively. Linearity ranges of syringin, eleutheroside E and isofraxidin were 2.46–492, 2.53–506, and 2.54–508 (μg/mL), respectively.

### 3.5. Static Adsorption and Desorption Tests

#### 3.5.1. Adsorption and Desorption Properties of the Resins

The static adsorption tests of the RAS extract were performed as follows: An amount of 0.5 g of resins (dry weight basis) was added to a conical flask with a lid and then 100 mL aqueous sample solutions with known concentrations prepared as described in section 3.3 were added. The flasks were then shaken (100 rpm) for 8 h at 25 °C in a constant temperature oscillator (Donglian, Heilongjiang, China). The initial concentration of the sample solutions and their concentrations post adsorption were analyzed by HPLC.

The static desorption process was carried out as follows: After reaching adsorption equilibrium, the residual solution was removed. The adsorbate-laden resins were first washed in 100 mL deionized water, shaken (100 rpm) for 2 h at 25 °C. Then, they were desorbed in 25 mL ethanol–water (95:5, v/v) solution. The flasks were shaken (100 rpm) for 2 h at 25 °C. Desorption solutions were also analyzed by HPLC. The suitable resin was selected based on its adsorption capacity, desorption capacity and desorption ratio.

#### 3.5.2. Static Adsorption Kinetics on HPD100C and HPD300

The static adsorption kinetics of syringin, eleutheroside E and isofraxidin on the preliminarily selected resins, HPD100C and HPD300, were also studied using the same procedure described in section 3.5.1. The initial concentrations of syringin, eleutheroside E and isofraxidin in the static adsorption kinetics experiments were 0.0077, 0.1090 and 0.0436 mg/mL, respectively. The respective concentrations of the three targeted components in the sample solutions were monitored by HPLC at predetermined time intervals until equilibrium.

#### 3.5.3. Adsorption Isotherms

The adsorption isotherms of syringin, eleutheroside E and isofraxidin on the optimum resin, HPD100C were investigated putting 100 mL of sample solutions at different concentrations in contact with pre-weighed resins weighing 0.5 g (dry weight basis) in the shaker bath (100 rpm) for 8 h at 25, 30 and 35 °C. The initial and equilibrium concentrations were determined by HPLC. The equilibrium adsorption isotherms on the resin were obtained, and their degrees of fitness to the Langmuir equation and Freundlich equation were evaluated.

### 3.6. Dynamic Adsorption and Desorption

Dynamic adsorption and desorption experiments were carried out in glass columns (12 mm × 500 mm) (Tianjin Tianbo Glass Instrument Co., Ltd., Tianjin, China) wet-packed with 5 g (dry weight basis) HPD100C. The bed volume (BV) and the length of the resin were 25 mL and 10 cm, respectively. In all cases, the sample solutions were flowed downward. Sample solutions were flowed through the glass column at a certain flow rate, and the concentrations of the three targeted components were monitored by HPLC analysis of the effluent liquid collected at 50 mL intervals. The breakthrough point was indicated.

Once adsorption reached equilibrium, the loading of the sample solution was stopped. The adsorbate-laden columns were first washed with deionized water and then with different ethanol–water solutions (30:70, 40:60, 50:50, 60:40, 70:30, 80:20, 90:10, v/v) at the same flow rate. The concentrations of the three targeted components in the effluent solution were determined by HPLC analysis of the effluent liquid collected at 5 mL intervals. The effluent solutions were concentrated and dried under vacuum before further analyses. The dynamic adsorption and desorption capacities of HPD100C, the recoveries and the contents of the three targeted components in the product were calculated.

### 3.7. Adsorption and Desorption Capacity, the Recovery Equation

The following equations were used to quantify the adsorption and desorption capacity, the desorption ratio as well as the recovery.

Adsorption evaluation:

(1) $$Q_{\text{e}}=(C_{0}-C_{\text{e}})\cdot \frac{V_{\text{i}}}{W}$$

where *Q*_e_ is the adsorption capacity at adsorption equilibrium (mg/g resin); *C*_0_ and *C*_e_ are the initial and equilibrium concentrations of solutes in the solutions, respectively (mg/mL); *V*_i_ is the volume of the initial sample solution (mL) and *W* is the weight of the dry resin (g).

Desorption evaluation

(2) $$D=\frac{C_{\text{d}}\cdot V_{\text{d}}}{(C_{0}-C_{\text{e}})\cdot V_{\text{i}}}\cdot 100\%$$

where *D* is the desorption ratio (%); *C*_d_ is the concentration of the solutes in the desorption effluent solutions(mg/mL); *V*_d_ is the volume of the desorption solutions; *C*_0_, *C*_e_ and *V*_i_ are the same as those defined in Equation (1).

The following equation was used to calculate the recovery.

(3) $$R=\frac{m}{M}\cdot 100\%$$

where *R* is the recovery (%), *M* is the weight of a targeted component laden onto the selected adsorbent, *m* is the weight of the targeted component in product.

### 3.8. Langmuir Equation and Freundlich Equation

The equilibrium experimental data were fitted to the Langmuir and Freundlich isotherm equations [27] to describe the adsorption behavior between solute and resin:

The Langmuir isotherm equation:

(4) $$\frac{C_{\text{e}}}{Q_{\text{e}}}=\frac{C_{\text{e}}}{Q_{\text{max}}}+\frac{1}{k\cdot Q_{\text{max}}}$$

The above equation can be rearranged to the following linear form:

(5) $$\frac{1}{Q_{\text{e}}}=\frac{1}{K_{\text{L}}\cdot C_{\text{e}}}+\frac{1}{Q_{\text{max}}}$$

where *Q*_max_ is the theoretically calculated maximum adsorption capacity (mg/g resin); *k* and *K*_L_ are constants; *Q*_e_ is the adsorption capacity at adsorption equilibrium (mg/g resin) and *C*_e_ is the equilibrium concentration of solutes in the solutions.

The Freundlich isotherm equation:

(6) $$Q_{\text{e}}=K_{\text{F}}\cdot C_{\text{e}}^{1/n}$$

A linearized form of Equation (6) can be written as:

(7) $$\text{log }Q_{\text{e}}=\text{log }K_{\text{F}}+\left(\frac{1}{n}\right)\cdot \text{log }C_{\text{e}}$$

where *K*_F_ is a constant, an indicator of adsorption capacity; 1/*n* is an empirical constant related to the magnitude of the adsorption driving force; *Q*_e_ and *C*_e_ are the same as those defined in Equations (4) and (5).

## 4. Conclusions

In this study, the preparative simultaneous separation of syringin, eleutheroside E and isofraxidin from RAS using macroporous resins was successfully achieved, and was demonstrated to be an approach with broad prospects. Among the 17 resins tested, HPD100C was selected, due to its higher surface area, optimum average pore diameter, appropriate surface functional polarity, and its absorption power resulting from hydrogen bonding and van der Waals forces. The equilibrium adsorption experiment of the three targeted components at 25 °C on HPD100C was fitted to the Langmuir isotherm model. In addition, the processes of dynamic adsorption and desorption were conducted to achieve the optimal separation parameters. Through the treatment on a column packed with HPD100C, the optimal adsorption and desorption conditions about simultaneous separation of three targeted components from RAS were obtained. After the treatment with HPD100C, the contents of syringin, eleutheroside E and isofraxidin improved 174-fold, 20-fold and 5-fold, respectively. The recoveries of the three targeted components were 80.93%, 93.97% and 93.79%, respectively. The results of this study will also serve the selection of macroporous resins for simultaneous preparative separation and purification of other phenol glycosides and aglucones from other herbal materials.

## Figures and Tables

**Table 1:** Adsorption capacities and desorption ratio of syringin, eleutheroside E and isofraxidin.

	Syringin		Eleutheroside E		Isofraxidin	
			
Resins	Adsorption capacity (mg/g)	Desorption ratio (%)	Adsorption capacity (mg/g)	Desorption ratio (%)	Adsorption capacity (mg/g)	Desorption ratio (%)
HPD100	0.24 ± 0.01	55.24 ± 2.71	6.32 ± 0.32	53.08 ± 2.72	4.59 ± 0.23	44.03 ± 2.19
HPD100B	0.20 ± 0.01	32.45 ± 1.42	5.76 ± 0.29	51.31 ± 2.60	3.96 ± 0.20	45.55 ± 2.25
HPD100C	0.42 ± 0.02	54.51 ± 2.72	5.82 ± 0.29	68.07 ± 3.42	3.76 ± 0.19	62.01 ± 3.11
HPD200A	0.23 ± 0.01	30.48 ± 1.58	5.80 ± 0.29	53.13 ± 2.59	4.02 ± 0.20	48.58 ± 2.29
HPD300	0.42 ± 0.02	51.54 ± 2.64	5.98 ± 0.30	64.77 ± 3.23	4.05 ± 0.20	57.15 ± 2.90
HPD700	0.11 ± 0.00	60.72 ± 3.12	3.94 ± 0.20	49.35 ± 2.45	4.25 ± 0.21	42.27 ± 2.19
HPDD	0.09 ± 0.00	50.24 ± 2.58	3.62 ± 0.18	46.40 ± 2.32	3.21 ± 0.16	51.98 ± 2.57
D101	0.13 ± 0.01	48.31 ± 2.54	4.42 ± 0.22	52.67 ± 2.57	3.23 ± 0.16	52.67 ± 2.49
HPD910	0.21 ± 0.01	24.39 ± 1.21	3.40 ± 0.17	53.55 ± 2.71	2.52 ± 0.13	66.57 ± 3.25
AB-8	0.05 ± 0.00	98.91 ± 4.63	5.33 ± 0.27	50.52 ± 2.63	3.23 ± 0.16	33.43 ± 1.46
HPD450	0.09 ± 0.00	50.90 ± 2.50	3.50 ± 0.18	48.61 ± 2.40	3.41 ± 0.17	44.53 ± 2.53
HPD750	0.09 ± 0.01	59.50 ± 2.93	3.15 ± 0.16	44.65 ± 2.22	3.44 ± 0.17	41.86 ± 2.23
HPD850	0.37 ± 0.02	37.48 ± 1.81	2.00 ± 0.10	57.60 ± 2.85	3.22 ± 0.16	56.74 ± 2.76
HPD400	0.21 ± 0.01	29.10 ± 1.58	4.68 ± 0.24	42.85 ± 2.06	4.33 ± 0.22	43.39 ± 2.23
HPD500	0.06 ± 0.00	99.66 ± 5.02	0.79 ± 0.04	43.71 ± 2.32	3.44 ± 0.17	61.27 ± 3.12
HPD600	0.11 ± 0.01	42.94 ± 2.23	1.20 ± 0.06	28.76 ± 1.43	3.95 ± 0.20	52.11 ± 2.57
HPD826	0.09 ± 0.01	58.33 ± 3.02	1.61 ± 0.08	38.77 ± 1.87	4.00 ± 0.20	52.75 ± 2.46

**Table 2:** Langmuir and Freundlich parameters of syringin, eleutheroside E and isofraxidin on HPC100C.

Adsorbate	Temperature (°C)	Langmuir equation			Freundlich equation		
	
		*Q*max	*K*_L_	*R*^2^	*K*_F_	*n*	*R*^2^
Syringin	25	0.52	1428.57	0.9922	0.9139	8.4746	0.9181
	30	0.48	833.33	0.9841	0.9543	6.5488	0.9309
	35	0.42	526.32	0.9718	0.9211	5.5928	0.9136
Eleutheroside E	25	10.79	769.23	0.9903	18.8930	3.5676	0.9201
	30	10.57	714.29	0.9891	18.8278	3.4211	0.9380
	35	10.33	526.32	0.9868	18.9758	3.0637	0.9393
Isofraxidin	25	6.33	434.78	0.9972	35.1965	1.7015	0.9622
	30	5.42	289.02	0.9857	38.9404	1.4943	0.9903
	35	4.79	208.33	0.9821	33.7054	1.4347	0.9942

**Table 3:** Effects of different ethanol**–**water solutions as desorption solutions on desorption properties of HPD100C for syringin, eleutheroside E and isofraxidin.

Ethanol–water solution (v/v)	30:70	40:60	50:50	60:40	70:30	80:20	90:10
Mass of dried residue (g)	2.00 ± 0.09	2.06 ± 0.11	2.09 ± 0.11	2.17 ± 0.10	2.38 ± 0.12	2.83 ± 0.10	3.50 ± 0.12
Mass of syringin (mg)	1.14 ± 0.05	1.28 ± 0.07	1.76 ± 0.06	2.10 ± 0.10	2.19 ± 0.10	2.26 ± 0.12	2.32 ± 0.12
Content of syringin (%)	0.057 ± 0.002	0.062 ± 0.003	0.084 ± 0.004	0.097 ± 0.004	0.092 ± 0.005	0.080 ± 0.004	0.066 ± 0.003
Mass of eleutheroside E (mg)	22.40 ± 1.06	25.67 ± 1.28	38.79 ± 1.89	46.09 ± 2.22	46.24 ± 2.28	46.38 ± 2.28	46.60 ± 2.25
Content of eleutheroside E (%)	1.118 ± 0.056	1.247 ± 0.062	1.854 ± 0.092	2.128 ± 0.110	1.943 ± 0.095	1.638 ± 0.083	1.333 ± 0.067
Mass of isofraxidin (mg)	12.54 ± 0.58	14.21 ± 0.73	21.18 ± 1.12	27.62 ± 1.39	27.71 ± 1.38	27.82 ± 1.42	27.98 ± 1.42
Content of isofraxidin (%)	0.626 ± 0.031	0.690 ± 0.035	1.012 ± 0.051	1.275 ± 0.055	1.165 ± 0.055	0.982 ± 0.048	0.801 ± 0.042

where mean ± S.D., n = 3.

**Table 4:** Contents and recoveries of syringin, eleutheroside E and isofraxidin separated on HPD100C.

Adsorbate	Content in untreated extract (%)	Content in product (%)	Recovery (%)
Syringin	0.04	6.97	80.93
Eleutheroside E	0.59	12.18	93.97
Isofraxidin	0.24	1.28	93.79

Untreated extract and product were dried at 60 °C for 12 h.

**Table 5:** Physical properties of the employed macroporous resins.

Resin	Surface area (m^2^/g)	Average pore diameter (Å)	Particle diameter (mm)	Polarity	Moisture content (%)
HPD100	650–700	85–90	0.300–1.200	Non-polar	65.00
HPD100B	500–580	120–160	0.300–1.250	Non-polar	61.49
HPD100C	720–760	80–90	0.300–1.250	Non-polar	61.68
HPD200A	700–750	85–90	0.300–1.250	Non-polar	54.90
HPD300	800–870	50–55	0.300–1.200	Non-polar	75.52
HPD700	650–700	85–90	0.300–1.200	Non-polar	66.10
HPDD	650–750	90–110	0.300–1.250	Non-polar	73.06
D101	≥400	100–110	0.300–1.250	Non-polar	66.47
HPD910	450–550	85–90	0.300–1.250	Non-polar	50.00
AB-8	480–520	130–140	0.300–1.250	Weak-polar	65.00
HPD450	500–550	90–110	0.300–1.200	Weak-polar	72.00
HPD750	650–700	85–90	0.300–1.200	Middle-polar	57.58
HPD850	1100–1300	85–95	0.300–1.200	Middle-polar	46.81
HPD400	500–550	75–80	0.300–1.200	Polar	68.93
HPD500	500–550	55–75	0.300–1.200	Polar	70.45
HPD600	550–600	80	0.300–1.200	Polar	69.32
HPD826	500–600	90–100	0.300–1.250	Polar	67.52
